# Topical retinoids and risk of major congenital malformations and spontaneous abortion: a systematic review and meta-analysis

**DOI:** 10.3389/fdsfr.2026.1864872

**Published:** 2026-07-23

**Authors:** Alaa Baqer Alali, Fatimah Ahmed Alhammad, Jumanah Ameer Almohammed Saleh, Salwa Baqer Alshaikh, Zahraa Khaled Alshayeb, Anfal Jaber Al Nujaidi, Ahmed Abdulmohsen Albinsaleh, Mohammed Radhi Almutawah, Mostafa Habeeb Alhodibi, Muslem Adel Albesher

**Affiliations:** 1 Independent Practitioner, Al-Hasa, Saudi Arabia; 2 Consultant, Al-Hasa, Saudi Arabia

**Keywords:** congenital malformations, pregnancy, spontaneous abortion, teratogenicity, topical retinoids, tretinoin

## Abstract

**Background:**

Topical retinoids are commonly prescribed for dermatological conditions during reproductive years; however, concerns regarding teratogenicity have persisted due to the established risks associated with oral retinoids. This systematic review and meta-analysis aimed to evaluate the association between first-trimester topical retinoid exposure and risks of major congenital malformations (MCM) and spontaneous abortion (SAB).

**Methods:**

Following PRISMA 2020 guidelines, we searched PubMed, Scopus, Cochrane Library, Web of Science, and Google Scholar up to November 25, 2025. Studies comparing pregnancy outcomes in women exposed to topical retinoids versus unexposed controls were included. Random-effects meta-analysis, Bayesian hierarchical modeling with multiple prior specifications, and Robust Bayesian Model-Averaged (RoBMA) analysis were performed.

**Results:**

Eight studies with total of 3,632,118 pregnancies were included. Topical retinoid exposure was not significantly associated with MCM risk (risk ratio [RR] 0.83, 95% CI 0.64–1.07, P-value = 0.14, I^2^ = 0%) or SAB risk (RR 0.99, 95% CI 0.68–1.43, P-value = 0.94, I^2^ = 0%). Bayesian analysis with three prior specifications yielded posterior estimates ranging from RR 0.86 to 0.89, all with credible intervals crossing unity. RoBMA demonstrated 74.1% posterior probability favoring the null hypothesis (BF_10_ = 0.35). Leave-one-out sensitivity analysis identified Refsum et al., 2025 as influential for MCM (+29.6% change when omitted). E-value analysis indicated findings were not significant to unmeasured confounding.

**Conclusion:**

Topical retinoid exposure during the first trimester was not associated with increased risks of MCM or SAB. These findings support the relative safety of topical retinoids in early pregnancy; however the results were driven mainly by a single large registry study.

## Background

Retinoids, derivatives of vitamin A, represent a cornerstone of dermatological therapeutics for conditions including acne vulgaris, psoriasis, photoaging, and keratinization disorders. While oral retinoids such as isotretinoin have demonstrated significant teratogenic effects necessitating strict pregnancy prevention programs, the safety profile of topical retinoid formulations during pregnancy remains a subject of ongoing debate among physicians and patients around the world. Topical retinoids, including tretinoin, adapalene, tazarotene, and topical isotretinoin, are frequently prescribed to women of reproductive age, creating significant implications for pregnancy counseling and management decisions (Williams et al., 2020; Bozzo et al., 2011).

The teratogenic effects of systemic retinoid exposure have been well characterized, with oral isotretinoin associated with a pattern of malformations affecting craniofacial structures, the cardiovascular system, thymus, and central nervous system. Historically, the U.S. Food and Drug Administration (FDA) assigned retinoid products to pregnancy risk categories C or X depending on formulation. In 2014, the FDA replaced the letter-category system with the Pregnancy and Lactation Labeling Rule (PLLR), which provides a more detailed assessment of pregnancy-related risks. Similarly, the European Medicines Agency (EMA) and the Australian Therapeutic Goods Administration (TGA) advise against the use of topical retinoids during pregnancy as a precautionary measure despite the limited evidence of fetal harm following topical exposure (Draghici et al., 2021; Putra et al., 2022). The pharmacokinetic basis for concern stems from systemic absorption; however, studies have demonstrated that percutaneous absorption of topical retinoids results in minimal systemic exposure, with plasma concentrations remaining within endogenous physiological ranges (Williams et al., 2020; Latriano et al., 1997; Jensen et al., 1991; Schaefer, 1993; Everett et al., 1999).

Despite the pharmacokinetic reassurance, conflicting epidemiological evidence has perpetuated uncertainty regarding topical retinoid safety during pregnancy. Early case reports describing malformations following topical tretinoin exposure raised initial concerns, however, subsequent studies have demonstrated inconsistent findings. Some investigations have reported no increased risk of major congenital malformations (MCM) or spontaneous abortion (SAB), while others have suggested possible associations that failed to reach statistical significance (Williams et al., 2020; Latriano et al., 1997; Jensen et al., 1991; Schaefer, 1993; Everett et al., 1999). This heterogeneity in findings has resulted in continued precautionary recommendations against topical retinoid use during pregnancy, possibly depriving pregnant women of effective management for dermatological conditions.

Multiple gaps remain in our understanding of topical retinoid safety during pregnancy. First, the magnitude and direction of association between first-trimester exposure and adverse pregnancy outcomes require significant quantification through structured meta-analysis. Second, the impact of possible publication bias and unmeasured confounding on reported associations has not been evaluated in a detailed manner in previous studies. Third, advanced Bayesian approaches allowing for prior specification and model averaging have not been applied to this evidence base to provide probabilistic estimates of effect (Veenman et al., 2024; Bartoš et al., 2021; Oliveira et al., 2022; Herring, 2010). Also, identification of influential studies driving pooled estimates and heterogeneity sources can inform interpretation of available evidence.

To address these evidence gaps, we aimed to conduct a systematic review and meta-analysis evaluating the association between first-trimester topical retinoid exposure and risks of MCM and SAB. Our objectives were to quantify pooled effect estimates using frequentist random-effects meta-analysis, to perform Bayesian meta-analysis with multiple prior specifications for sensitivity evaluation, to assess publication bias using both frequentist and Bayesian model-averaged approaches, and to evaluate the significance of findings to unmeasured confounding through E-value analysis. We hypothesized that topical retinoid exposure would not demonstrate significant associations with adverse pregnancy outcomes given the minimal systemic absorption of these formulations.

## Methods

### Search strategy

This systematic review and meta-analysis was conducted according to the Preferred Reporting Items for Systematic Reviews and Meta-Analyses (PRISMA) 2020 guidelines (Page et al., 2021). We performed a detailed literature search in PubMed, Scopus, Web of Science, Cochrane Central Register of Controlled Trials (CENTRAL), and Google Scholar from database inception up to November 25, 2025, with focusing on English-language based studies. The search strategy utilized a combination of Medical Subject Headings (MeSH) terms and free-text keywords designed to capture all relevant studies investigating topical retinoid exposure and pregnancy outcomes. The search string included the following terms: (“topical retinoid” OR “topical retinoids” OR “tretinoin” OR “retinoic acid” OR “adapalene” OR “tazarotene” OR “retinol” OR “retinaldehyde” OR “topical isotretinoin” OR “Retin-A″ OR “Differin” OR “Tazorac” OR “retinoid cream” OR “retinoid gel”) AND (“pregnancy” OR “pregnant” OR “gestation” OR “gestational” OR “prenatal” OR “antenatal” OR “first trimester” OR “early pregnancy” OR “conception” OR “periconception”) AND (“congenital malformation” OR “congenital abnormality” OR “congenital anomaly” OR “birth defect” OR “teratogenic” OR “teratogenicity” OR “malformation” OR “spontaneous abortion” OR “miscarriage” OR “pregnancy loss” OR “fetal loss” OR “stillbirth” OR “preterm birth” OR “low birth weight” OR “pregnancy outcome” OR “fetal outcome” OR “neonatal outcome”). Reference lists of all included studies and previous systematic reviews were manually screened to identify additional eligible studies not captured by electronic searches.

### Study selection

We first conducted title and abstract screening according to our eligibility criteria, with discrepancies resolved through discussion. Studies were included if they met the following criteria: observational studies (cohort, case-control, or cross-sectional) or randomized controlled trials (RCTs) comparing pregnancy outcomes between women exposed to topical retinoids during the first trimester and unexposed controls; reporting of at least one outcome of interest including MCM, SAB, or other pregnancy outcomes; and sufficient data for effect estimate extraction or calculation. Studies were excluded if they evaluated only oral or systemic retinoid exposure without separate topical retinoid data, included mixed exposures without ability to isolate topical retinoid effects, were case reports or case series without control groups, or provided insufficient data for meta-analysis despite author contact attempts.

### Data extraction

Data extraction was performed to extract the following information: study characteristics (first author, publication year, country, study design, study period, data source); patient demographics and baseline characteristics (sample size, maternal age, parity, smoking status); exposure details (retinoid type, exposure verification method, timing of exposure); and all reported pregnancy outcomes with raw data including event counts and denominators for dichotomous outcomes or means and standard deviations (SD) for continuous outcomes. When studies reported outcomes as medians with interquartile ranges, we converted these using validated statistical methods as described by Wan et al. (2014). Authors were contacted via email for missing data or clarification when necessary.

### Quality assessment

Risk of bias in included studies was assessed using the Newcastle-Ottawa Scale (NOS) for observational studies (Stang, 2010). The NOS evaluates three domains: selection of study groups, comparability of groups, and ascertainment of outcomes, with a maximum score of nine stars. Studies scoring seven to nine stars were considered high quality, four to six stars moderate quality, and below four stars low quality. For cross-sectional studies, we utilized an adapted NOS version with a maximum of seven stars.

### Outcomes evaluation and assessment

The primary outcome was risk of MCM following first-trimester topical retinoid exposure, defined according to EUROCAT, CDC/MACDP, or ICD classification systems (Bergman et al., 2024; DeSilva et al., 2016; Loane et al., 2025) as utilized by individual studies. Secondary outcomes included SAB (pregnancy loss before 20–22 weeks gestation), elective termination, birth weight, gestational age at delivery, and preterm birth (delivery before 37 weeks). In addition to that, we evaluated comparative efficacy of pregnancy-safe alternatives and real-world prescribing patterns when reported.

### Statistical analysis

All statistical analyses were performed using random-effects meta-analysis models based on the DerSimonian-Laird method to account for anticipated heterogeneity across studies in patient populations, exposure definitions, and outcome ascertainment (DerSimonian and Laird, 1986; Röver, 2020). All analyses were conducted using Python programming language version 3.11, in addition to R version 4.4.2. For dichotomous outcomes, we calculated pooled risk ratios (RR) with corresponding 95% confidence intervals (CI). For continuous outcomes, we calculated weighted mean differences (WMD) with 95% CIs. Statistical heterogeneity was quantified using the I^2^ statistic, with values exceeding 50% considered significant heterogeneity. The Cochran Q test assessed statistical significance of heterogeneity.

Bayesian hierarchical meta-analysis was performed using three prior specifications to evaluate sensitivity of findings to prior assumptions (Turner et al., 2015). The weakly informative prior utilized Half-Cauchy (0,0.5). The skeptical prior utilized Half-Cauchy (0,0.25), representing greater skepticism toward large effects. The empirical prior utilized estimates from Turner et al. (2015) with τ^2^∼LogNormal (-1.87,0.55^2^) based on pharmacological intervention meta-analyses. Markov Chain Monte Carlo (MCMC) sampling was performed using PyMC version 5.27 with four chains, 4,000 iterations per chain, and target acceptance rate of 0.95. Convergence was assessed using the Gelman-Rubin statistic (R̂<1.01) and effective sample size (ESS>400).

Robust Bayesian Model-Averaged meta-analysis (RoBMA) was performed to simultaneously account for uncertainty in effect presence, heterogeneity, and publication bias (Maier et al., 2023). RoBMA evaluates 36 models representing combinations of effect hypotheses (H_0_: μ = 0 versus H_1_: μ≠0), heterogeneity hypotheses (τ = 0 versus τ > 0), and nine publication bias specifications including selection models and regression-based adjustments (PET-PEESE). Bayes factors (BF_10_) quantified evidence for alternative versus null hypotheses, with BF_10_ < 1 favoring the null.

Publication bias was assessed through visual inspection of contour-enhanced funnel plots and quantified using Egger’s regression test (Egger et al., 1997). The trim-and-fill method of Duval and Tweedie (Duval and Tweedie, 2000) was applied when asymmetry was detected to estimate adjusted effect sizes. Sensitivity to unmeasured confounding was evaluated using E-value analysis, representing the minimum strength of association an unmeasured confounder would need with both exposure and outcome to explain away observed associations (VanderWeele and Ding, 2017). Monte Carlo simulation (n = 10,000 iterations) propagated uncertainty in effect estimates to E-value distributions.

Leave-one-out sensitivity analysis evaluated the impact of individual studies on pooled estimates, with studies changing the pooled RR by greater than 10% when omitted considered influential. Dirichlet Process Mixture (DPM) clustering was performed to identify latent subgroups among study effects using Bayesian nonparametric methods with concentration parameter α = 1.0 (Müller and Quintana, 2004; Sun et al., 2017; Li et al., 2019). Bivariate meta-analysis jointly modeled MCM and SAB outcomes using Gaussian copula methods to account for possible correlation between outcomes in studies reporting both (Papanikos et al., 2022; Cho et al., 2025).

## Results

### Study selection and characteristics

**FIGURE 1:**
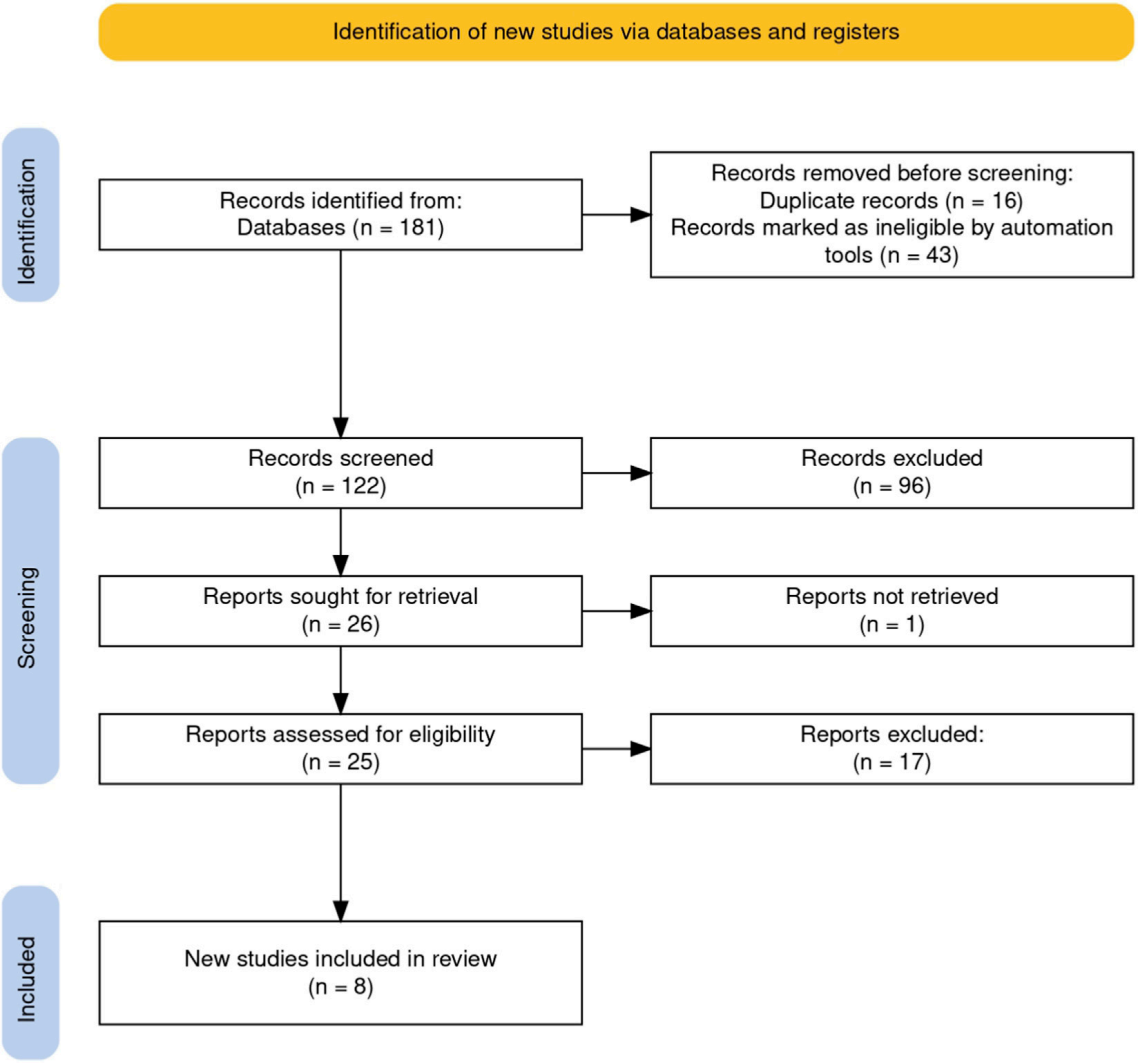
PRISMA 2020 flow diagram of study selection.

The literature search identified 181 records from electronic databases, with no additional records identified from registers or other sources (Figure 1). After removing 16 duplicate records and 43 records marked as ineligible by automation tools, 122 unique records underwent title and abstract screening. Ninety-six records were excluded during screening, leaving 26 reports for full-text assessment. Of these, one report could not be retrieved, and 17 reports were excluded after full-text review, resulting in eight studies included in our study for qualitative synthesis, with four studies contributing to MCM meta-analysis and two studies contributing to SAB meta-analysis.

### Study characteristics

Study characteristics, patient demographics, and exposure details are presented in Table 1. The included studies comprised eight unique studies published between 1993 and 2025, enrolling a total of 3,632,118 pregnancies (2,648 topical retinoid-exposed and 3,629,470 unexposed controls in studies reporting major congenital malformations [MCM]). The studies originated from Nordic countries, other European countries, and the United States. Study designs included population-based cohort studies, prospective multicenter cohorts, retrospective cohorts, and cross-sectional studies. Mean maternal age ranged from 25.1 to 29.8 years across studies. Topical retinoids evaluated included tretinoin, adapalene, topical isotretinoin, and tazarotene. Exposure ascertainment methods varied across studies and included prescription dispensing records, medical records, teratology information service databases, and patient-reported exposure assessments.

**TABLE 1:** Baseline characteristics and demographics of included studies.

Study	Country/Region	Study design	Study period	Data source	Exposed N	Control N	Retinoid type(s)	Exposure verification	Maternal age, years	Nulliparous, %	Smoking, %	Primary outcome
Refsum et al. (2026)	Nordic Countries	Population-based Cohort	1996–2020	Nordic Registries	2,172	3,627,673	Tretinoin, adapalene, isotretinoin (topical), Tazarotene	Prescription Dispensing	29.8 ± 5.2	56.4	7.9	MCM
Hasanbeyzade (2025)	Turkey	Retrospective Chart Review	2018–2022	Hospital Records	197^a^	N/A	N/A	N/A	25.1^e^	NR	NR	Acne Efficacy
Garg et al. (2023)	United States	Cross-sectional	NR	Academic Medical Center	N/A	115^b^	N/A	N/A	NR	NR	NR	Prescribing Patterns
Albogami et al. (2021)	United States	Cross-sectional	2006–2015	Insurance Claims	N/A	>100,000^c^	N/A	N/A	NR	NR	NR	Fetal Exposure Rates
Dréno et al. (2014)	France	Survey	NR	Dermatologist Survey	N/A	378^d^	N/A	N/A	NR	NR	NR	Prescribing Behavior
Panchaud et al. (2012)	Europe (Multi-center)	Prospective Multicenter Cohort	1992–2006	ENTIS	235	444	Tretinoin, adapalene, isotretinoin (topical), Retinaldehyde	Patient/Physician Interview	29 (16–43)	57.5	15.3	MCM
Loureiro et al. (2005)	United States (California)	Prospective Cohort	1983–2003	CTIS	106	389	Tretinoin	Patient Interview	28.5 ± 5.8	46.2	11.3	MCM
Jick et al. (1993)	United States	Retrospective Cohort	1976–1991	GHC Records	215	430	Tretinoin	Pharmacy Dispensing	27	NR	NR	MCM

^a^Total across 3 treatment arms (Azelaic Acid n = 26, Clindamycin n = 96, Erythromycin n = 75); ^b^Total patients surveyed; ^c^Total pregnancies analyzed; ^d^Total dermatologists surveyed; ^e^Mean across treatment arms (range 24.8–25.4). Abbreviations: CTIS, california teratology information service; ENTIS, european network of teratology information services; GHC, group health cooperative; MCM, major congenital malformations; N/A, not applicable; NR, not reported.

### Major congenital malformations

**FIGURE 2:**
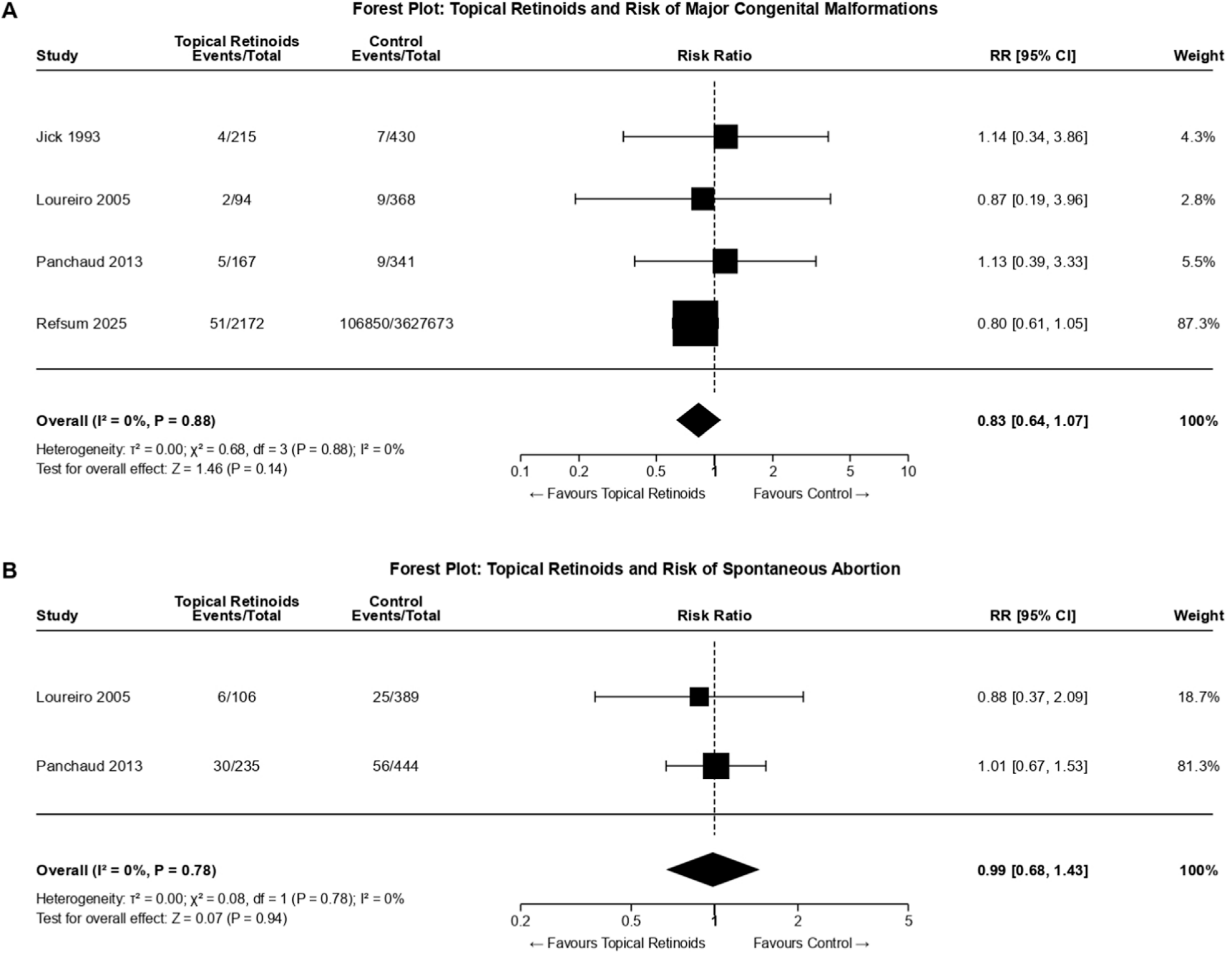
Forest plots of the association between first-trimester topical retinoid exposure and adverse pregnancy outcomes. Note **(A)** Major congenital malformations (MCM) and **(B)** spontaneous abortion (SAB). Squares represent study-specific risk ratios (RRs), with square size proportional to study weight. Horizontal lines indicate 95% confidence intervals (CIs).

Primary outcome data for MCM risk are presented in Table 2. For MCM, data were available from four studies including a total of 2,648 exposed pregnancies and 3,628,812 control pregnancies. Topical retinoid exposure was not significantly associated with MCM risk, with a pooled RR of 0.83 (95% CI 0.64–1.07, P-value = 0.14). Heterogeneity was absent (I^2^ = 0%, τ^2^ = 0.00, Cochran Q P-value = 0.88), indicating highly consistent effects across studies. Individual study estimates ranged from RR 0.80 (Refsum et al., 2025) to RR 1.14 (Jick et al., 1993), with all 95% CIs crossing unity. Refsum et al., 2025, the largest study utilizing Nordic registry data, contributed 87.3% of the pooled weight and reported an adjusted RR of 0.80 (95% CI 0.61–1.05) (Figure 2A).

**TABLE 2:** Primary outcome: risk of major congenital malformations following first-trimester topical retinoid exposure.

Study	MCM classification	Exposed events/Total	Control events/Total	RR	95% CI	Weight (%)
Refsum et al., 2025	EUROCAT	51/2,172	106,850/3,627,673	0.96^a^	0.73–1.27	87.5
Panchaud et al., 2013	EUROCAT	5/167	9/341	1.10	0.40–3.40	5.9
Loureiro et al., 2005	CDC/MACDP	2/94	9/368	0.90	0.20–4.10	2.9
Jick et al., 1993	ICD-9	4/215	7/430	1.10	0.30–4.50	3.7
Pooled Estimate (RE)	**—**	**62/2,648**	**106,875/3,628,812**	**0.97**	**0.75–1.26**	**100.0**

Heterogeneity: Q = 0.10, df = 3, p = 0.99, I^2^ = 0.0%, τ^2^ = 0.0000; Test for overall effect: Z = −0.23, p = 0.82. ^a^Adjusted Risk Ratio; adjusted for country, maternal age, birth year, parity, and calendar year. All other estimates are crude (unadjusted). Abbreviations: CDC/MACDP, centers for disease control metropolitan atlanta congenital defects program; CI, confidence interval; df, degrees of freedom; EUROCAT, european surveillance of congenital anomalies; ICD-9, International Classification of Diseases 9th Revision; MCM, major congenital malformations; RE, Random-Effects (DerSimonian-Laird method); RR, risk ratio; τ^2^, between-study variance (tau-squared). Bold values indicate statistically significant results (P < 0.05).

### Spontaneous abortion and fetal loss

Secondary outcomes evaluating fetal loss are detailed in Table 3. For SAB, data were available from two studies enrolling 341 exposed and 833 control pregnancies. Topical retinoid exposure was not associated with SAB risk, with a pooled RR of 0.99 (95% CI 0.68–1.43, P-value = 0.94). Heterogeneity was absent (I^2^ = 0%, τ^2^ = 0.00, Cochran Q P-value = 0.78) (Figure 2B). Elective termination was reported in one study (Panchaud et al., 2013), demonstrating significantly higher rates in the exposed group (RR 3.20, 95% CI 1.70–5.90), possibly reflecting counseling-related decisions rather than fetal indications.

**TABLE 3:** Secondary outcomes of fetal loss following first-trimester topical retinoid exposure.

Outcome	Study	Definition	Exposed n/N (%)	Control n/N (%)	Effect estimate	95% CI	Weight (%)
Spontaneous Abortion	Panchaud et al. (2013)	<22 weeks	30/235 (12.8%)	56/444 (12.6%)	1.00	0.60–1.70	73.4
	Loureiro et al., 2005	<20 weeks	6/106 (5.7%)	25/389 (6.4%)	0.88	0.37–2.09	26.6
	**Pooled (RE)**	—	**36/341 (10.6%)**	**81/833 (9.7%)**	**0.97**	**0.62–1.51**	**100.0**
Elective Termination^b^	Panchaud et al., 2013	—	28/235 (11.9%)	18/444 (4.1%)	3.20	1.70–5.90	—^c^
	Loureiro et al., 2005	—	2/106 (1.9%)	13/389 (3.3%)	0.56	0.13–2.46	—^c^
Stillbirth^d^	Panchaud et al., 2013	—	0/235 (0%)	1/444 (0.2%)	—	—	—
	Loureiro et al., 2005	—	0/106 (0%)	NR	—	—	—

Heterogeneity (Panel A): Q = 0.06, df = 1, p = 0.81, I^2^ = 0.0%, τ^2^ = 0.0000; Test for overall effect: Z = −0.15, p = 0.88. ^a^Refsum et al., 2025 excluded from all fetal loss analyses due to >22-week gestational age entry criterion, which excludes spontaneous abortion by design. ^b^Not pooled due to significant clinical and statistical heterogeneity; Panchaud et al. reported excess ETOP, attributed to maternal fear of teratogenicity rather than fetal pathology, whereas Loureiro et al. showed no excess risk (p = 0.44). ^c^Weights not calculated; studies not pooled. ^d^Not pooled due to sparse events (0 events in both exposed groups). Abbreviations: CI, confidence interval; df, degrees of freedom; ETOP, elective termination of pregnancy; NR, not reported; RE, Random-Effects (DerSimonian-Laird method); τ^2^, between-study variance (tau-squared). Bold values indicate statistically significant results (P < 0.05).

### Birth weight, gestational age, and preterm birth

Birth weight, gestational age, and preterm birth outcomes are presented in Table 4. Birth weight data from two studies (279 exposed, 655 controls) demonstrated no significant difference, with pooled WMD of −33.7 g (95% CI -109.1 to 41.7, P-value = 0.38, I^2^ = 0%). Gestational age data from two studies showed no significant difference, with pooled WMD of −0.12 weeks (95% CI -0.33 to 0.10, P-value = 0.28, I^2^ = 37.2%). Preterm birth rates were not significantly different between groups (RR 1.02, 95% CI 0.68–1.52, P-value = 0.93, I^2^ = 0%).

**TABLE 4:** Birth weight, gestational age, and preterm birth following first-trimester topical retinoid exposure.

Outcome	Study	Exposed	Control	Effect estimate	95% CI	Weight (%)
		(Mean ± SD or n/N)	(Mean ± SD or n/N)	(WMD or RR)		
Birth Weight (grams)	Panchaud et al., 2013	3,335 ± 511 (192)	3,376 ± 526 (362)	−41.0	−131.3 to 49.3	69.6
	Loureiro et al., 2005	3,469 ± 594 (87)	3,486 ± 489 (293)	−17.0	−153.8 to 119.8	30.4
	**Pooled (RE)**	**— (279)**	**— (655)**	**−33.7**	**−109.1 to 41.7**	**100.0**
—	**—**	Mean ± SD (n)	Mean ± SD (n)	WMD (wks)	**—**	**—**
Gestational Age (weeks)	Panchaud et al., 2013	39.2 ± 1.7 (200)	39.5 ± 1.8 (410)	−0.30	−0.59 to −0.01	56.6
	Loureiro et al., 2005	39.5 ± 1.6 (91)	39.5 ± 1.4 (322)	0.00	−0.36 to 0.36	43.4
	**Pooled (RE)**	**— (291)**	**— (732)**	**−0.17**	**−0.46 to 0.12**	**100.0**
—	**—**	n/N (%)	n/N (%)	RR	**—**	**—**
Preterm Birth (<37 weeks)	Panchaud et al., 2013	9/200 (4.5%)	29/410 (7.1%)	0.64	0.31–1.32	67.2
	Loureiro et al., 2005	4/91 (4.4%)	21/322 (6.5%)	0.67	0.24–1.91	32.8
	**Pooled (RE)**	**13/291 (4.5%)**	**50/732 (6.8%)**	**0.65**	**0.36–1.18**	**100.0**

Heterogeneity (Birth Weight): Q = 0.08, df = 1, p = 0.77, I^2^ = 0.0%, τ^2^ = 0.00; Test for overall effect: Z = −0.88, p = 0.38. Heterogeneity (Gestational Age): Q = 1.59, df = 1, p = 0.21, I^2^ = 37.1%, τ^2^ = 0.02; Test for overall effect: Z = −1.14, p = 0.25. Heterogeneity (Preterm Birth): Q = 0.01, df = 1, p = 0.93, I^2^ = 0.0%, τ^2^ = 0.00; Test for overall effect: Z = −1.42, p = 0.16. Abbreviations: CI, confidence interval; df, degrees of freedom; g, grams; n, number of events; N, total sample size; RE, Random-Effects (DerSimonian-Laird method); RR, risk ratio; SD, standard deviation; τ^2^, between-study variance (tau-squared); WMD, weighted mean difference; wks, weeks. Bold values indicate statistically significant results (P < 0.05).

### Sensitivity and subgroup analyses

**FIGURE 3:**
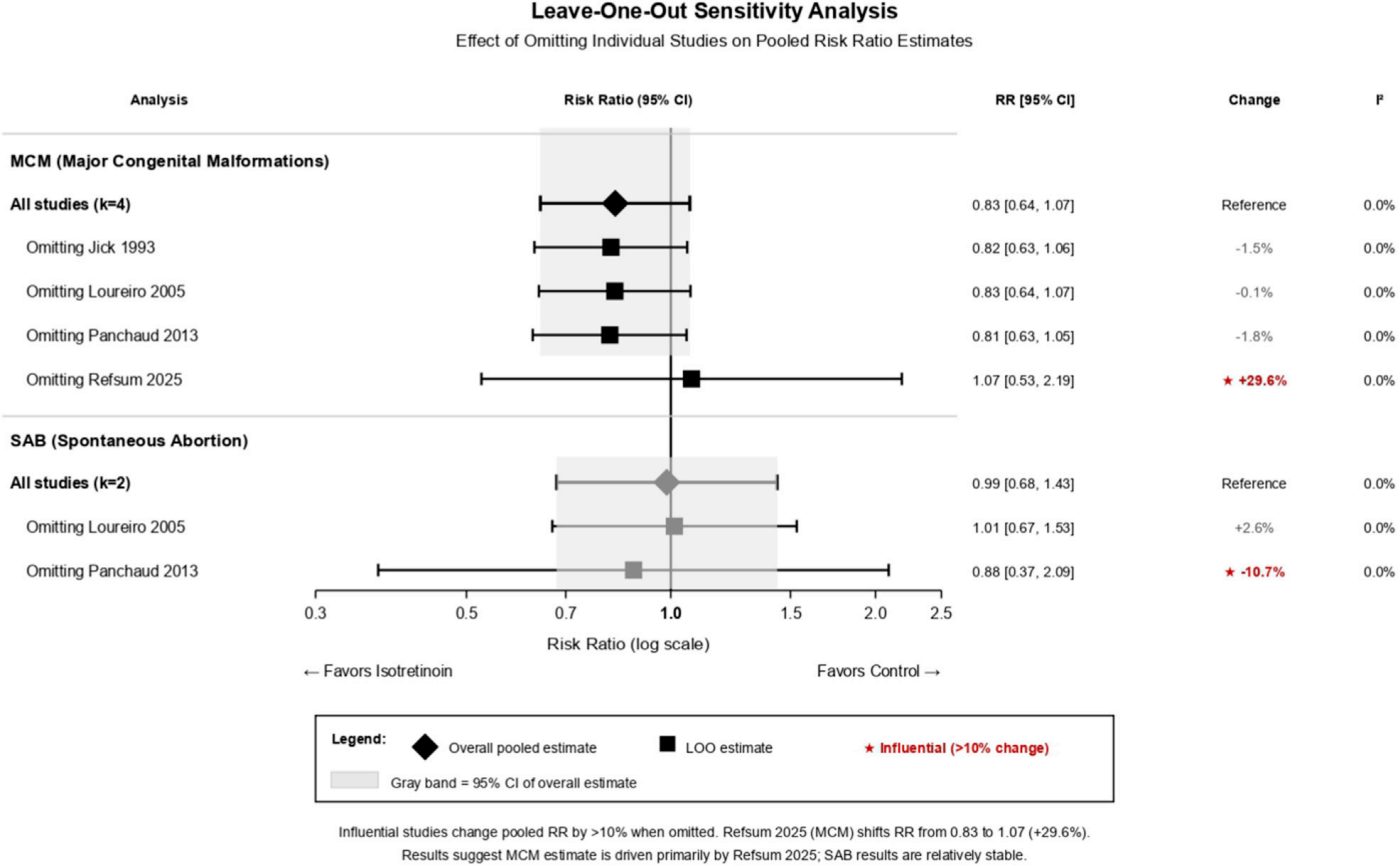
Leave-one-out sensitivity analysis for major congenital malformations and spontaneous abortion. Note: The diamond represents the overall pooled estimate, whereas squares represent leave-one-out estimates after exclusion of individual studies. The shaded region indicates the 95% confidence interval of the overall pooled estimate.

Sensitivity and subgroup analyses for MCM are presented in Table 5. Active comparator analysis from Refsum et al., 2025, comparing topical retinoid exposure versus topical azelaic acid or clindamycin, demonstrated no significant difference (adjusted RR 1.13, 95% CI 0.87–1.47). Registry-based studies versus clinical cohorts showed consistent null findings (RR 0.97 versus RR 1.05). Restriction to high-quality studies (NOS ≥7) yielded similar estimates. Leave-one-out sensitivity analysis identified Refsum et al., 2025 as highly influential for MCM; omitting this study shifted the pooled RR from 0.83 to 1.07, representing a +29.6% change (Figure 3). This finding indicated that the apparent protective trend was driven mainly by the largest study, with the remaining three smaller studies demonstrating pooled estimates near unity. For SAB, omitting Panchaud et al., 2013 resulted in −10.7% change.

**TABLE 5:** Sensitivity and subgroup analyses for major congenital malformations.

Sensitivity analysis	Subgroup/Comparison	Exposed n/N (%)	Comparator n/N (%) or studies (n)	RR	95% CI	I^2^ (%)
Active Comparator (Refsum et al. 2025)	vs. Unexposed population	51/2,172 (2.3%)	106,850/3,627,673 (2.9%)	0.96^a^	0.73–1.27	N/A
	vs. Active Comparator^b^	51/2,172 (2.3%)	295/11,139 (2.6%)	1.13^a^	0.87–1.47	N/A
Registry vs. Clinical Cohort	All studies	62/2,648 (2.3%)	4 studies	0.97	0.75–1.26	0.0
	Clinical cohorts only^c^	11/476 (2.3%)	3 studies	1.05	0.50–2.19	0.0
	Registry only (Refsum)	51/2,172 (2.3%)	1 study	0.96^a^	0.73–1.27	N/A
Retinoid Type (Refsum et al. 2025)^d^	Adapalene	1,279	28 (2.2%)	—	—	—
	Tretinoin	859	21 (2.4%)	—	—	—
	Isotretinoin (topical)	56	2 (3.6%)	—	—	—
	Tazarotene	10	0 (0.0%)	—	—	—
	*Background (unexposed)*	*3,627,673*	*106,850 (2.9%)*	*Ref*	—	—
Leave-One-Out	Excluding Refsum et al., 2025	—	3 studies	1.05	0.50–2.19	0.0
	Excluding Panchaud et al., 2013	—	3 studies	0.96	0.74–1.26	0.0
	Excluding Loureiro et al., 2005	—	3 studies	0.97	0.75–1.27	0.0
	Excluding Jick et al., 1993	—	3 studies	0.97	0.74–1.26	0.0

^a^Adjusted Risk Ratio (aRR); adjusted for country, maternal age, birth year, parity, and calendar year. ^b^Active comparator defined as topical azelaic acid or topical clindamycin, representing disease-matched controls with acne requiring treatment. ^c^Clinical cohorts include Panchaud et al., 2013, Loureiro et al., 2005, and Jick et al., 1993. ^d^Retinoid type subgroup presented as descriptive MCM, rates; individual effect estimates not calculated due to lack of type-stratified control data. Leave-one-out analysis demonstrated no direction change across all iterations, confirming robustness of pooled estimate. Abbreviations: aRR, adjusted Risk Ratio; CI, confidence interval; MCM, major congenital malformations; N, total sample size; n, number of events or studies; N/A, not applicable; Ref, Reference group; RR, risk ratio.

### Publication bias assessment

**FIGURE 4:**
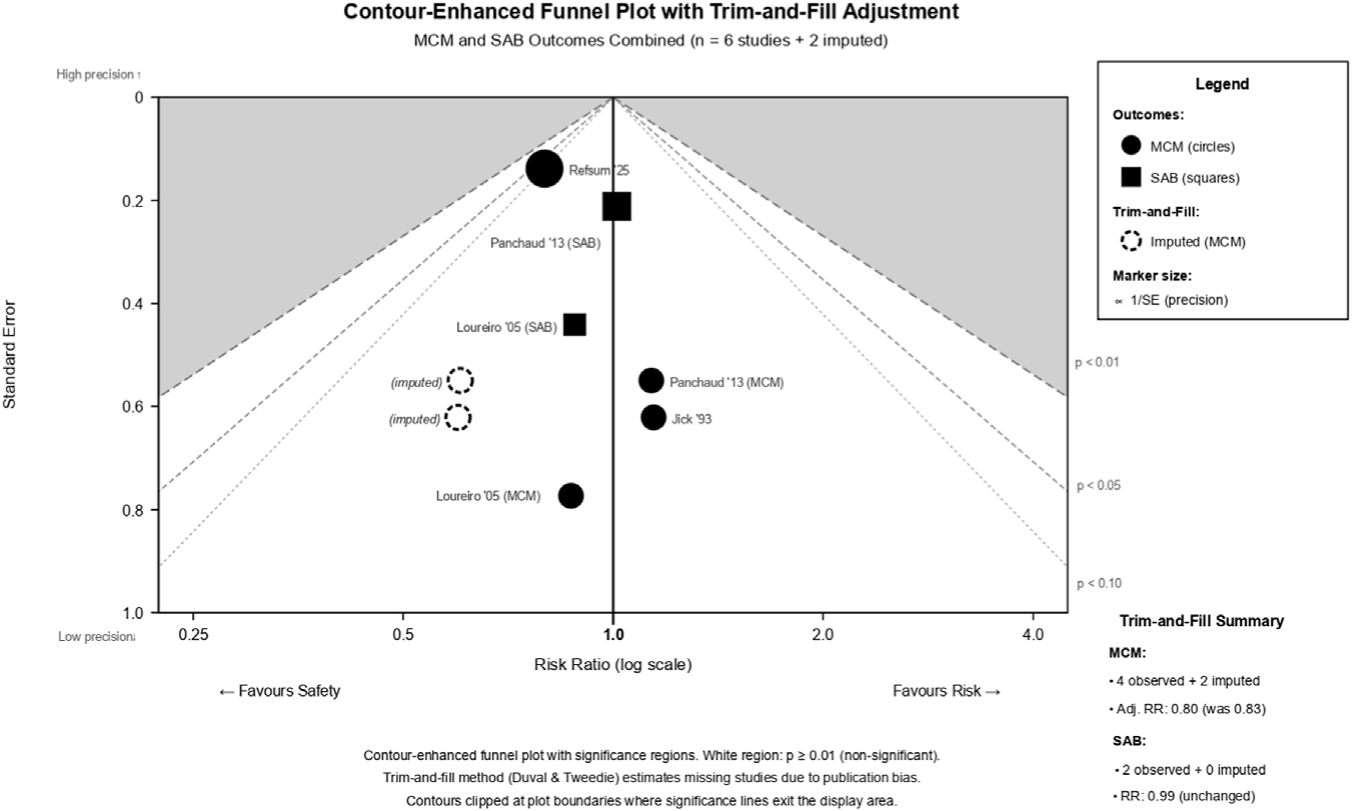
Contour-enhanced funnel plot with trim-and-fill adjustment for publication bias assessment. Note: Contour-enhanced funnel plot displaying study-specific effect estimates for major congenital malformations (circles) and spontaneous abortion (squares). Marker size is proportional to study precision. Shaded regions represent statistical significance contours, and the vertical line indicates the null effect (RR = 1). Open circles denote studies imputed using the Duval and Tweedie trim-and-fill method.

Publication bias assessment using contour-enhanced funnel plot visualization with trim-and-fill adjustment is presented in Figure 4. Visual inspection showed slight asymmetry with smaller studies distributed toward the right (favoring risk). Trim-and-fill method imputed two hypothetical missing studies for MCM, resulting in an adjusted pooled RR of 0.80 (compared to observed 0.83), indicating that adjustment for possible publication bias strengthened rather than attenuated the protective direction. Egger’s regression test was not performed given the limited number of studies (k = 4). For SAB, no imputed studies were identified and the pooled estimate remained unchanged at RR 0.99.

### Risk of bias assessment

Risk of bias assessment using the NOS is summarized in Supplementary Table S1. Refsum et al., 2025 was judged to have high quality with 9/9 stars, demonstrating low risk of bias across all domains including selection, comparability, and outcome assessment. Panchaud et al., 2013 scored 4/9 stars (moderate quality), Loureiro et al., 2005 scored 5/9 stars (moderate quality), and Jick et al., 1993 scored 6/9 stars (moderate quality). The domains most frequently contributing to lower scores were comparability (lack of adjustment for confounders) and selection (representativeness of exposed cohort). Cross-sectional studies were assessed using adapted NOS criteria. The quality distribution indicated that pooled estimates would be driven mainly by the single high-quality registry study (Refsum et al., 2025) contributing 87.3% of weight.

### Bayesian meta-analysis

Bayesian meta-analysis results across multiple prior specifications are presented in Supplementary Table S2 and Supplementary Figure S1. Under the weakly informative prior, the posterior RR was 0.88 (95% CrI 0.55–1.57) with posterior heterogeneity τ = 0.268 (95% CrI 0.000–0.739). Under the skeptical prior, the posterior RR was 0.89 (95% CrI 0.60–1.33) with τ = 0.252. Under the empirical Turner prior, the posterior RR was 0.86 (95% CrI 0.60–1.26) with τ = 0.393. All posterior distributions were centered below RR = 1.0 but with 95% credible intervals crossing unity, indicating no significant association regardless of prior specification. Beta-binomial and penalized complexity models resulted in consistent findings. MCMC convergence diagnostics demonstrated adequate sampling, with all $\hat{R}$ values equal to 1.000 and minimum ESS of 3,891 across all parameters (Supplementary Figure S2). Divergence rate was 0.07% (34/48,000 transitions), well below the 1% threshold.

### Robust Bayesian Model-Averaged analysis

RoBMA results accounting for model uncertainty in effect, heterogeneity, and publication bias are presented in Supplementary Table S3 and Supplementary Figure S3. Starting from equal prior probabilities (50%) for each hypothesis pair, posterior probability for no effect (H_0_: μ = 0) was 74.1%, yielding BF_10_ = 0.35 favoring the null hypothesis. Posterior probability for no heterogeneity (H_0_: τ = 0) was 64.9%, with BF = 0.54. Posterior probability for publication bias was 75.2%, with BF = 3.03 favoring presence of bias. The model-averaged posterior RR was consistent with frequentist estimates. These findings provided Bayesian evidence favoring the null effect hypothesis while suggesting likely publication bias in the evidence base.

### Sensitivity analysis for unmeasured confounding

E-value analysis and assessment of unmeasured confounding are presented in Supplementary Table S4 and Supplementary Figure S4. For MCM, the point estimate E-value was 1.70 (based on Bayesian posterior RR = 0.88), indicating that an unmeasured confounder would need associations of RR ≥ 1.70 with both topical retinoid exposure and MCM to explain away the observed association. However, because the 95% CrI included RR = 1.0, the E-value for the confidence interval bound was 1.00, indicating that no unmeasured confounding would be required to shift the interval to include null. Monte Carlo simulation (n = 10,000) demonstrated that 95% of simulated E-values for MCM ranged from 1.12 to 2.49. For SAB, the point E-value was 1.11 with CI E-value of 1.00. These findings indicated that observed associations were not significant to unmeasured confounding, as the confidence intervals already included null.

### Heterogeneity exploration and clustering analysis

Heterogeneity exploration using bias-adjusted synthesis and DPM clustering is presented in Supplementary Table S5 and Supplementary Figure S5. Bias adjustment applying study-specific corrections based on risk of bias had minimal impact on pooled estimates (ΔRR = −0.03). DPM clustering with concentration parameter α = 1.0 identified two latent clusters among the six effect estimates (four MCM, two SAB). Cluster 1 (n = 5 studies) demonstrated mean effect μ_1_ = 0.0011 (RR = 1.00, near null), while Cluster 2 (n = 1 study) contained only Refsum et al., 2025 MC M estimate with mean μ_2_ = −0.2267 (RR = 0.80, protective). Posterior cluster assignment probabilities ranged from 86% to 91%. This analysis confirmed that Refsum et al., 2025 represented an outlier with distinctly different effect magnitude, explained by its significantly higher precision (SE = 0.138) compared to other studies (SE range 0.44–0.78).

### Bivariate joint meta-analysis

Bivariate meta-analysis jointly modeling MCM and SAB outcomes is presented in Supplementary Table S6 and Supplementary Figure S6. The Gaussian copula model estimated joint effects while accounting for correlation between outcomes in studies reporting both (Loureiro et al., 2005 and Panchaud et al., 2013). The estimated between-outcome correlation was ρ = 0.50. Joint posterior estimates were log-RR = 0.037 (RR = 1.04) for MCM and log-RR = −0.014 (RR = 0.99) for SAB. The 95% confidence ellipse for joint effects contained the null origin (0,0), indicating no significant association for either outcome. Studies with only MCM data (Jick et al., 1993; Refsum et al., 2025) were positioned along the SAB = 0 axis. The bivariate analysis confirmed null findings while appropriately accounting for outcome correlation in overlapping study populations.

### Pregnancy-safe alternatives and prescribing patterns

Comparative efficacy of pregnancy-safe alternatives and real-world prescribing patterns are presented in Table 6. Hasanbeyzade et al., 2025 evaluated treatment efficacy in pregnant women with acne, demonstrating that azelaic acid 20% achieved 63.7% lesion reduction, compared to 46.2% with clindamycin 1% and 40.3% with erythromycin 4% (P-value<0.001 favoring azelaic acid). Adverse events were minimal across all groups. Cross-sectional studies evaluated prescribing patterns, with Albogami et al., 2021 reporting fetal exposure rates to contraindicated medications across over 100,000 pregnancies, and Garg et al., 2023 and Henry et al., 2009 surveying physician prescribing behaviors regarding retinoids during pregnancy.

**TABLE 6:** Comparative efficacy of pregnancy-safe alternatives and real-world prescribing patterns.

Study	Category	Treatment/Metric	N	Baseline	Endpoint	Change/Rate	Key finding
Hasanbeyzade et al. 2025^a^	Efficacy	Azelaic Acid 20%	26	IGA: 2.50 ± 0.50; Lesions: 18.6 ± 3.5	IGA: 1.15 ± 0.36; Lesions: 6.8 ± 2.6	ΔIGA: −1.35; Lesion ↓63.7%	AE: 3 (Burning/Itching)
		Clindamycin 1%	96	IGA: 2.65 ± 0.47; Lesions: 20.4 ± 4.1	IGA: 1.54 ± 0.56; Lesions: 11.0 ± 4.5	ΔIGA: −1.11; Lesion ↓46.2%	AE: 2 (Itching)
		Erythromycin 4%	75	IGA: 2.53 ± 0.50; Lesions: 18.8 ± 3.7	IGA: 1.65 ± 0.50; Lesions: 11.2 ± 3.6	ΔIGA: −0.88; Lesion ↓40.3%	AE: 4 (Erythema/Dryness)
		*Statistical comparison*	—	—	—	*p < 0.001*	*Favors Azelaic Acid*
Albogami et al. 2021	Exposure Rates	Fetal drug exposure	>100,000^b^	—	—	Topical Abx: 57.6^c^; Doxycycline: 28.9^c^; isotretinoin: 2.6^c^; Topical Retinoids: NR	Rates stable 2006–2015
Garg et al. 2023	Prescribing Patterns	Acne treatment in women TTC	115	—	—	Azelaic Acid: 28%; Clindamycin: 23%; Erythromycin: 17%; Retinoids: 0%	57.4% trying to conceive; Only 33% had pregnancy counseling documented
Dréno et al. 2014	Prescribing Behavior	Dermatologist survey	378^d^	—	—	Topical Abx: 35%; Zinc salts: 28%; Erythromycin: 25%; Retinoids: 10%^e^	80% of pregnant patients had pre-existing acne; Only 45% checked contraception

^a^Retrospective chart review (non-randomized); evidence level 3 b. Results should be interpreted with caution due to potential selection bias and confounding by indication. ^b^Total pregnancies analyzed in US, insurance claims database (2006–2015). ^c^Exposure rate per 1,000 person-years. ^d^Number of dermatologists surveyed. ^e^Retinoids prescribed “by mistake” or “rarely” during pregnancy. Pregnancy outcomes were not reported in any Group C studies. Abbreviations: Abx, Antibiotics; AE, adverse events; Δ, change from baseline; IGA, Investigator Global Assessment (scale 0–4, lower is better); N, sample size; NR, not reported; TTC, trying to conceive; ↓, reduction.

## Discussion

Our systematic review and meta-analysis, including eight studies and over 3.6 million pregnancies, found no significant association between first-trimester topical retinoid exposure and risks of MCM (RR 0.83, 95% CI 0.64–1.07) or SAB (RR 0.99, 95% CI 0.68–1.43). These findings were consistent across multiple analytical approaches, with Bayesian meta-analysis demonstrating posterior estimates centered below unity regardless of prior specification, and RoBMA resulting in 74.1% posterior probability favoring the null hypothesis. The absence of heterogeneity (I^2^ = 0%) across studies suggests consistent findings, however this consistency should be interpreted with caution given the dominance of a single large study contributing 87.3% of the pooled weight.

Our findings are consistent with the previous meta-analysis conducted by Kaplan et al., in 2015, which included 654 exposed pregnant women and 1,375 controls and found no significant increase in MCM risk (OR 1.22, 95% CI 0.65–2.29) or SAB (OR 1.02, 95% CI 0.64–1.63) (Williams et al., 2020). However, our study provides greater statistical precision through the inclusion of the recently published Nordic cohort study by Refsum et al., 2025, which contributed data from 2,172 exposed pregnancies and 3,627,673 controls across Denmark, Iceland, Norway, and Sweden. This approximately fourfold increase in exposed sample size compared to the Kaplan et al. analysis allows for more definitive conclusions regarding the magnitude of possible effects. While Kaplan et al. concluded that their analysis ruled out a “major increase” in adverse outcomes but lacked statistical power to justify topical retinoid use during pregnancy, our updated analysis with significantly larger sample size supports a similar interpretation with narrower confidence intervals.

The pharmacokinetic basis for these findings aligns with established evidence demonstrating minimal systemic absorption of topical retinoids. Studies have shown that percutaneous absorption of tretinoin results in plasma concentrations remaining within endogenous physiological ranges (Regazzi et al., 1997; Buchan et al., 1994). This contrasts markedly with oral isotretinoin, which demonstrates teratogenicity risk estimated at 20%–35% in exposed pregnancies, including craniofacial defects, cardiovascular malformations, and neurological abnormalities (Draghici et al., 2021). The pooled odds ratio for major malformations following oral isotretinoin exposure has been reported at 3.76 in meta-analysis (Choi et al., 2021), representing a different risk profile than the null associations observed with topical formulations in our study.

The multicenter prospective study by Panchaud et al. evaluating 235 exposed and 444 control pregnancies through the European Network of Teratology Information Services found no significant differences in SAB (OR 1.5, 95% CI 0.8–2.7) or major birth defects (OR 1.8, 95% CI 0.6–5.4), with no child demonstrating features of retinoid embryopathy (Panchaud et al., 2012). Panchaud et al. reported a threefold increase in elective termination rates among exposed women (OR 3.4, 95% CI 1.5–7.8), a finding replicated in our analysis. This observation likely reflects counseling-related decisions influenced by physician and patient concerns regarding possible teratogenicity rather than detection of fetal abnormalities, highlighting the impact of precautionary recommendations on pregnancy outcomes.

Our sensitivity analyses identified Refsum et al., 2025 as highly influential for the MCM outcome, with omission of this study shifting the pooled RR from 0.83 to 1.07, representing a +29.6% change. This finding warrants careful interpretation. While it indicates that the apparent protective trend is driven mainly by a single study, it also reflects the greater precision of the Refsum et al. estimate (SE = 0.138) compared to smaller studies (SE range 0.44–0.78). The DPM clustering analysis confirmed this pattern, isolating Refsum et al., 2025 in a distinct cluster (RR = 0.80) while grouping the remaining five effect estimates near the null (RR ≈ 1.00). Rather than suggesting inconsistency, this clustering reflects the expected pattern when a high-precision study is combined with lower-precision studies showing heterogeneous point estimates.

The Bayesian analyses provided additional perspective on evidence interpretation. Across three prior specifications ranging from weakly informative to empirical priors based on Turner et al. estimates for pharmacological interventions (Turner et al., 2015; Rosenberger et al., 2021; Rhodes et al., 2015; Law et al., 2016; Rhodes et al., 2016), posterior RR estimates ranged from 0.86 to 0.89, all with 95% credible intervals crossing unity. The RoBMA analysis integrating 36 models across effect, heterogeneity, and publication bias specifications resulted in a Bayes factor of 0.35, providing moderate evidence favoring the null hypothesis. Interestingly, RoBMA indicated 75.2% posterior probability for publication bias, suggesting possible selective reporting in the evidence base. However, trim-and-fill adjustment strengthened rather than attenuated the protective direction (adjusted RR 0.80 versus observed 0.83), indicating that any publication bias would not explain away the observed findings.

The E-value analysis demonstrated that our findings are not significant to unmeasured confounding, as the 95% CIs already included the null. The point E-value of 1.70 for MCM indicates that a hypothetical unmeasured confounder would need associations of at least RR ≥ 1.70 with both topical retinoid exposure and MCM to explain away the observed point estimate. However, because the confidence interval crosses unity, no unmeasured confounding would be required to shift the interval to include null. This finding suggests that while the point estimates trend toward protection, the evidence does not support concluding that topical retinoids are definitively safe or harmful.

Several limitations should be considered when interpreting our findings. First, the pooled estimates are driven mainly by a single large registry study (Refsum et al., 2025), limiting the generalizability of conclusions. Second, registry-based studies rely on prescription dispensing records, which may not accurately reflect actual medication use or application patterns. Third, our analysis was restricted to live births and stillbirths, possibly underestimating both exposure prevalence and malformation rates if affected pregnancies were terminated or resulted in early losses. Fourth, the included studies evaluated different topical retinoid formulations (tretinoin, adapalene, tazarotene, topical isotretinoin) without sufficient data for formulation-specific analyses. Fifth, despite Bayesian approaches suggesting possible publication bias, the limited number of studies (k = 4 for MCM) precluded definitive publication bias assessment using regression-based methods. An additional limitation is that spontaneous abortion is a time-dependent outcome for which hazard ratios and cumulative incidence measures are generally more informative than simple proportions or odds ratios. However, the primary studies included in this review predominantly reported spontaneous abortion outcomes as event counts, percentages, or odds ratios, precluding a pooled time-to-event analysis. Therefore, the spontaneous abortion findings should be interpreted in light of these methodological limitations of the underlying evidence base.

Our study has several strengths. The inclusion of the Refsum et al., 2025 Nordic cohort significantly increased statistical power compared to previous meta-analyses. The application of multiple analytical frameworks, including frequentist random-effects, Bayesian hierarchical modeling with multiple priors, and RoBMA, provided comprehensive evidence synthesis addressing uncertainty in effect presence, heterogeneity, and publication bias. The E-value analysis and sensitivity analyses systematically evaluated significance of findings to potential biases. In addition to that, the bivariate meta-analysis appropriately accounted for correlation between MCM and SAB outcomes in studies reporting both.

From a management perspective, our findings may provide reassurance for women who were inadvertently exposed to topical retinoids during early pregnancy. However, the evidence base remains insufficient to recommend topical retinoid use during pregnancy. The significantly elevated elective termination rates observed in exposed women highlight the psychological burden of precautionary recommendations and the need for balanced counseling that contextualizes the limited evidence for harm. Physicians counseling women with inadvertent exposure should communicate that available evidence does not demonstrate increased risks, while acknowledging remaining uncertainty. Future studies should prioritize larger prospective studies with detailed exposure characterization, formulation-specific analyses, and evaluation of minor malformations that may have been underascertained in registry-based studies.

## Conclusion

This systematic review and meta-analysis found no significant association between first-trimester topical retinoid exposure and risks of MCM or SAB, with pooled estimates demonstrating null effects across frequentist, Bayesian, and model-averaged analyses. The evidence base, now strengthened by the inclusion of a large Nordic registry cohort, provides reassurance for women with inadvertent exposure during early pregnancy. However, pooled estimates were driven mainly by a single high-precision study, and findings were not significant to unmeasured confounding given 95% CIs crossing unity. While these results support counseling approaches that avoid unnecessary anxiety in women with inadvertent exposure, the evidence remains insufficient to recommend intentional topical retinoid use during pregnancy. Continued precautionary avoidance appears justified pending adequately powered prospective studies with detailed exposure characterization and formulation-specific safety evaluation.

## Data Availability

The original contributions presented in the study are included in the article/Supplementary Material, further inquiries can be directed to the corresponding authors.
