# Supplementary material for: Topical retinoids and risk of major congenital malformations and spontaneous abortion: a systematic review and meta-analysis
Source: Front Drug Saf Regul. 2026 Jul 23;6:1864872. doi: 10.3389/fdsfr.2026.1864872 (PMC13445963; doi:10.3389/fdsfr.2026.1864872)
Supplement: Supplementary file 1 [file Supplementaryfile1.docx]

**Supplementary Figures S1:** Bayesian Posterior Distribution Plot For Pooled Effect Sizes

**Supplementary Figures S2:** MCMC Convergence Diagnostics Plot

**Supplementary Figures S3:** RoBMA Component Analysis Plot

**Supplementary Figures S4:** E-Values Senstivity Analysis Plot

**Supplementary Figures S5:** Dirichlet Process Mixture Clustering Plot

**Supplementary Figures S6:** Bivariate Joint Meta-Analysis Plot
