# Supplementary material for: Topical retinoids and risk of major congenital malformations and spontaneous abortion: a systematic review and meta-analysis
Source: Front Drug Saf Regul. 2026 Jul 23;6:1864872. doi: 10.3389/fdsfr.2026.1864872 (PMC13445963; doi:10.3389/fdsfr.2026.1864872)
Supplement: Supplementary file 2 [file Table1.docx]

**Supplementary Table 1:** Risk of Bias Assessment of The Included Studies.

| **Study** | **Study Design** | **RoB Tool** | **S1** | **S2** | **S3** | **S4** | **C1** | **C2** | **O1** | **O2** | **O3** | **Selection** | **Comparability** | **Outcome** | **Total Score** | **Quality** |
| --- | --- | --- | --- | --- | --- | --- | --- | --- | --- | --- | --- | --- | --- | --- | --- | --- |
| Refsum et al. 2025 | Population-based Cohort | NOS | ★ | ★ | ★ | ★ | ★ | ★ | ★ | ★ | ★ | 4/4 | 2/2 | 3/3 | 9/9 | High |
| Panchaud et al. 2012 | Prospective Multicenter Cohort | NOS | ☆ | ★ | ☆ | ★ | ☆ | ☆ | ☆ | ★ | ★ | 2/4 | 0/2 | 2/3 | 4/9 | Moderate |
| Loureiro et al. 2005 | Prospective Cohort | NOS | ☆ | ★ | ☆ | ★ | ☆ | ☆ | ★ | ★ | ★ | 2/4 | 0/2 | 3/3 | 5/9 | Moderate |
| Jick et al. 1993 | Retrospective Cohort | NOS | ★ | ★ | ★ | ★ | ☆ | ☆ | ☆ | ★ | ★ | 4/4 | 0/2 | 2/3 | 6/9 | Moderate |
| Hasanbeyzade et al. 2025 | Retrospective Chart Review | NOS | ☆ | ☆ | ★ | ★ | ☆ | ☆ | ☆ | ★ | ★ | 2/4 | 0/2 | 2/3 | 4/9 | Moderate |
| Albogami et al. 2021 | Cross-sectional Database Analysis | NOS-CSᵃ | ★ | ★ | ★ | ★ | ☆ | — | ★ | ★ | — | 4/4 | 0/1 | 2/2 | 6/7 | Moderate-High |
| Garg et al. 2023 | Cross-sectional Chart Review | NOS-CSᵃ | ☆ | ☆ | ★ | ★ | ☆ | — | ★ | ★ | — | 2/4 | 0/1 | 2/2 | 4/7 | Moderate |
| Dréno et al. 2014 | Cross-sectional Survey | NOS-CSᵃ | ☆ | ★ | ☆ | ☆ | ☆ | — | ☆ | ★ | — | 1/4 | 0/1 | 1/2 | 2/7 | Low |

***Notes:*** *ᵃNOS-CS: Adapted Newcastle-Ottawa Scale for Cross-Sectional Studies (maximum 7 stars); items C2 and O3 not applicable.* ***NOS Domain Items — Cohort Studies:*** *S1, Representativeness of exposed cohort; S2, Selection of non-exposed cohort; S3, Ascertainment of exposure; S4, Outcome not present at start; C1, Comparability (main confounder adjusted); C2, Comparability (additional confounders adjusted); O1, Assessment of outcome; O2, Adequate follow-up length; O3, Adequacy of follow-up (attrition).* ***NOS Domain Items — Cross-Sectional Studies:*** *S1, Representativeness of sample; S2, Sample size adequacy; S3, Non-respondent handling; S4, Exposure/variable ascertainment; C1, Comparability/confounding control; O1, Outcome assessment; O2, Statistical analysis appropriateness.* ***Quality Thresholds (Cohort):*** *High, 7–9 stars; Moderate, 4–6 stars; Low, 0–3 stars.* ***Quality Thresholds (Cross-Sectional):*** *Moderate-High, 6–7 stars; Moderate, 4–5 stars; Low, 0–3 stars. ★, criterion met (1 point); ☆, criterion not met (0 points); —, not applicable.* ***Abbreviations:*** *C, Comparability domain; ETOP, Elective Termination of Pregnancy; HMO, Health Maintenance Organization; IGA, Investigator Global Assessment; NOS, Newcastle-Ottawa Scale; NOS-CS, Newcastle-Ottawa Scale adapted for Cross-Sectional studies; O, Outcome domain; RoB, Risk of Bias; S, Selection domain; TIS, Teratology Information Service.*

**Supplementary Table 2:** Bayesian Meta-Analysis Results for Major Congenital Malformations (k=4 studies).

| **Model** | **Prior Specification** | **Prior Distribution** | **Pooled RR** | **95% CrI** | **τ** | **95% CrI (τ)** |
| --- | --- | --- | --- | --- | --- | --- |
| Bayesian Hierarchical | Weakly Informative | μ ~ N(0, 1²); τ ~ Half-Cauchy(0, 0.5) | 0.88 | 0.53–1.48 | 0.268 | 0.000–0.739 |
|  | Skeptical | μ ~ N(0, 0.5²); τ ~ Half-Cauchy(0, 0.5) | 0.89 | 0.58–1.42 | 0.252 | 0.000–0.695 |
|  | Empirical (Turner) | μ ~ N(0, 1²); τ² ~ LogN(-1.66, 0.85²) | 0.92 | 0.52–1.66 | 0.393 | 0.137–0.697 |
| Beta-Binomial | Standard | logit(μ) ~ N(-3.66, 1); κ ~ Gamma(2, 0.1) | 1.00 | 0.33–3.02 | 0.345 | 0.000–0.984 |
|  | Conservative | logit(μ) ~ N(-3.66, 0.5); κ ~ Gamma(1, 0.05) | 1.03 | 0.48–2.21 | 0.248 | 0.000–0.715 |
| INLA | PC Prior (U=0.5, α=0.5) | PC prior: P(τ > 0.5) = 0.5 | 0.88 | 0.54–1.55 | 0.290 | 0.000–0.820 |
|  | PC Prior (U=0.3, α=0.05) | PC prior: P(τ > 0.3) = 0.05 | 0.84 | 0.62–1.14 | 0.088 | 0.000–0.252 |
|  | PC Prior (U=1.0, α=0.5) | PC prior: P(τ > 1.0) = 0.5 | 0.89 | 0.54–1.63 | 0.327 | 0.000–0.929 |

***Abbreviations:*** *CrI, credible interval; INLA, integrated nested Laplace approximation; κ, concentration/overdispersion parameter; LogN, log-normal distribution; N, normal distribution; PC, penalized complexity; RR, risk ratio; τ, between-study heterogeneity standard deviation; U, threshold parameter; α, tail probability.*

**Supplementary Table 3:** Robust Bayesian Model-Averaged Meta-Analysis (RoBMA) for Publication Bias Assessment.

| **Analysis Component** | **Parameter/Model** | **Prior/Distribution** | **Estimate** | **Result/Interpretation** |
| --- | --- | --- | --- | --- |
| Model Ensemble | Total models evaluated | — | 36 | 2 (effect) × 2 (heterogeneity) × 9 (publication bias) |
|  | Effect hypotheses | H₀: μ=0; H₁: μ≠0 | Prior P(H₁) = 0.50 | Equal prior probability for null and alternative |
|  | Heterogeneity hypotheses | τ=0 (fixed); τ>0 (random) | Prior P(τ>0) = 0.50 | Equal prior probability for fixed and random effects |
|  | Publication bias models | None + 6 selection + PET + PEESE | 9 specifications | Comprehensive bias adjustment coverage |
| Prior Specifications | Effect size (H₁) | μ ~ Normal(0, 1) | — | Weakly informative, centered on null |
|  | Heterogeneity | τ ~ Half-Cauchy(0, 0.5) | — | Allows small to moderate heterogeneity |
|  | PET coefficient | β_PET ~ Normal(0, 1) | — | Tests precision-effect relationship |
|  | PEESE coefficient | β_PEESE ~ Normal(0, 5) | — | Tests precision-effect with SE² |
|  | Selection weight (ω) | ω ~ Beta(1, 1) | — | Uniform prior on selection probability |
| Individual Models | Fixed effect, no bias | μ ~ N(0, 1); τ = 0 | RR = 0.80 | Pooled using inverse-variance weights |
|  | Random effects, no bias | μ ~ N(0, 1); τ ~ HC(0, 0.5) | RR = 0.88 | τ = 0.249 |
|  | Random effects + PET | Adds β_PET × SE term | RR = 0.83 | β_PET = 0.252 (no bias detected) |
|  | Random effects + PEESE | Adds β_PEESE × SE² term | RR = 0.84 | β_PEESE = 0.420 (no bias detected) |
|  | Selection model (p<0.05) | ω = selection weight | RR = 0.88 | ω = 0.50 (no selective reporting) |
| Bayes Factors | Effect inclusion (BF₁₀) | Savage-Dickey ratio | 0.284 | Moderate evidence for null (BF₁₀ < 1/3) |
|  | Heterogeneity inclusion (BF_τ) | Posterior mass ratio | 2.630 | Weak evidence for heterogeneity |
|  | Publication bias (BF_bias) | Model comparison | <1.0 | No evidence of publication bias |
| Publication Bias Tests | Studies with p<0.05 | — | 0 of 4 | No significant individual studies |
|  | PET test (H₀: β=0) | Small-study effects | β = 0.25 | No evidence of bias (\|β\| < 0.5) |
|  | PEESE test (H₀: β=0) | Small-study effects | β = 0.42 | No evidence of bias (\|β\| < 1.0) |
|  | Egger regression intercept | Funnel asymmetry | NS | Symmetric funnel plot expected |
| Model-Averaged Results | Pooled RR | Weighted across 36 models | 0.86 | 95% CrI: 0.56–1.47 |
|  | Heterogeneity (τ) | Posterior mean | 0.249 | Low to moderate heterogeneity |
|  | Posterior P(H₀) | Model probability | 0.78 | High probability of null effect |
|  | Posterior P(no bias) | Model probability | >0.80 | Publication bias unlikely |

***Abbreviations:*** *BF, Bayes factor; BF₁₀, Bayes factor for alternative vs null hypothesis; BF_τ, Bayes factor for heterogeneity; CrI, credible interval; H₀, null hypothesis; H₁, alternative hypothesis; HC, Half-Cauchy distribution; N, normal distribution; NS, not significant; PET, precision-effect test; PEESE, precision-effect estimate with standard error; RoBMA, robust Bayesian model-averaged meta-analysis; RR, risk ratio; SE, standard error; τ, between-study heterogeneity standard deviation; ω, selection model weight parameter.*

**Supplementary Table 4:** Sensitivity Analyses for Unmeasured Confounding and Systematic Bias.

| **Analysis Component** | **Parameter** | **Value** | **Distribution/Range** | **Result** | **Interpretation/Source** |
| --- | --- | --- | --- | --- | --- |
| E-Value Analysis | Point estimate | RR = 0.88 | — | E-value = 1.53 | Confounder needs RR ≥1.53 with both exposure and outcome |
|  | 95% CrI bound | RR = 1.00ᵃ | — | E-value = 1.00 | No unmeasured confounding needed to shift CI to null |
| Confounder Assessment | Maternal age >35 years | RR with MCM: 1.3–1.5 | — | Insufficient | Weak association with topical retinoid use (age-related acne decline) |
|  | Pre-gestational diabetes | RR with MCM: 2.0–4.0 | — | Insufficient | No expected association with topical retinoid use |
|  | Obesity (BMI ≥30 kg/m²) | RR with MCM: 1.3–1.5 | — | Insufficient | Weak possible association with topical retinoid use |
|  | Smoking during pregnancy | RR with MCM: 1.2–1.5 | — | Insufficient | No expected association with topical retinoid use |
|  | Folic acid deficiency | RR with MCM: 1.5–2.0 | — | Insufficient | No expected association with topical retinoid use |
|  | Alcohol use in pregnancy | RR with MCM: 1.2–1.5 | — | Insufficient | No expected association with topical retinoid use |
| Bias Parameters (n=100,000) | Selection bias (TIS studies) | OR adjustment | Trapezoidal (0.85, 0.90, 0.95, 1.00) | — | Beck 2017; Johnson 2001 |
|  | Selection bias (Registry studies) | OR adjustment | Trapezoidal (0.95, 0.98, 1.02, 1.05) | — | Population-based design |
|  | MCM sensitivity (Registry) | Proportion | Trapezoidal (0.85, 0.88, 0.92, 0.95) | — | EUROCAT validation |
|  | MCM sensitivity (TIS) | Proportion | Trapezoidal (0.80, 0.83, 0.87, 0.90) | — | Expert judgment |
|  | MCM specificity (All studies) | Proportion | Trapezoidal (0.99, 0.995, 0.998, 1.00) | — | High specificity assumption |
|  | Exposure sensitivity (Registry) | Proportion | Trapezoidal (0.85, 0.88, 0.92, 0.95) | — | Prescription records |
|  | Exposure sensitivity (TIS) | Proportion | Trapezoidal (0.70, 0.77, 0.83, 0.90) | — | Self-report validation |
|  | Exposure specificity (All studies) | Proportion | Trapezoidal (0.95, 0.97, 0.98, 0.99) | — | High specificity assumption |
| Bias-Adjusted Results | Combined adjustment | Median RR = 0.96 | 95% SI: 0.91–1.02 | 0.0% sims RR >1.5 | Selection + misclassification bias |
|  | Selection bias only | Median RR = 0.83 | 95% SI: 0.81–0.86 | 0.0% sims RR >1.5 | TIS healthy-user bias adjustment |
|  | Misclassification only | Median RR = 0.95 | 95% SI: 0.91–1.00 | 0.0% sims RR >1.5 | Exposure + outcome misclassification |

***Notes:*** *ᵃ 95% CrI includes 1.0; therefore, E-value for confidence interval bound = 1.00.* ***Abbreviations:*** *BMI, body mass index; CI, confidence interval; CrI, credible interval; E-value, evidence value for unmeasured confounding; EUROCAT, European Surveillance of Congenital Anomalies; MCM, major congenital malformations; OR, odds ratio; RR, risk ratio; SI, simulation interval; sims, simulations; TIS, Teratology Information Service.*

**Supplementary Table 5:** Heterogeneity Exploration Using Generalized Evidence Synthesis with Bias Adjustment and Dirichlet Process Mixture Model Clustering.

| **Analysis Component** | **Parameter/Study** | **Design/Prior** | **Risk of Bias** | **Value/Estimate** | **Result (95% CrI)** |
| --- | --- | --- | --- | --- | --- |
| Bias-Adjusted Synthesis | Refsum 2025 | Registry, adjusted | Low | Bias: 0.00 ± 0.05 | Adj. RR = 0.81 |
|  | Panchaud 2013 | TIS, unadjusted | Moderate | Bias: 0.10 ± 0.15 | Adj. RR = 0.87 |
|  | Loureiro 2005 | TIS, unadjusted | Moderate | Bias: 0.10 ± 0.15 | Adj. RR = 0.84 |
|  | Jick 1993 | Cohort, unadjusted | Moderate-High | Bias: 0.15 ± 0.20 | Adj. RR = 0.87 |
|  | Pooled (unadjusted) | Standard RE model | — | RR = 0.88 | 0.54–1.48 |
|  | Pooled (bias-adjusted) | Bias-adjusted RE model | — | RR = 0.85 | 0.52–1.40 |
|  | Heterogeneity (τ) | τ ~ Half-Cauchy(0, 0.5) | — | τ = 0.251 | Low-moderate heterogeneity |
|  | Change due to adjustment | ΔRR | — | ΔRR = -0.03 | Minimal change after bias adjustment |
| DPM Clustering | Concentration (α) | α ~ Gamma(1, 1) | — | α = 1.02 | 0.00–3.04 |
|  | Expected clusters | E[K] ≈ α × log(n) | — | E[K] = 1.4 | For n=4, α=1.02 |
|  | Within-cluster τ | τ ~ Half-Cauchy(0, 0.5) | — | τ = 0.277 | 0.000–0.765 |
|  | Effective clusters | Weight > 0.10 | — | K = 3 | Studies cluster together |
|  | Cluster 1 mean | μ ~ Normal(0, 1) | — | RR = 0.97 (P = 0.32) | 0.20–4.99 |
|  | Cluster 2 mean | μ ~ Normal(0, 1) | — | RR = 0.94 (P = 0.34) | 0.18–3.92 |
|  | Cluster 3 mean | μ ~ Normal(0, 1) | — | RR = 0.97 (P = 0.34) | 0.21–4.28 |
|  | Refsum 2025 | Observed RR = 0.80 | — | Cluster 3 | P(C1)=0.32, P(C2)=0.33, P(C3)=0.34 |
|  | Panchaud 2013 | Observed RR = 1.13 | — | Cluster 3 | P(C1)=0.32, P(C2)=0.33, P(C3)=0.35 |
|  | Loureiro 2005 | Observed RR = 0.87 | — | Cluster 3 | P(C1)=0.32, P(C2)=0.34, P(C3)=0.34 |
|  | Jick 1993 | Observed RR = 1.14 | — | Cluster 3 | P(C1)=0.31, P(C2)=0.34, P(C3)=0.36 |

***Notes:*** *Bias adjustment had minimal impact on pooled estimates (ΔRR = -0.03), with unadjusted and adjusted results nearly identical; DPM clustering revealed a homogeneous pattern with all studies clustering together and no distinct subgroups identified; overall, results were robust and stable across both bias adjustment and clustering analyses, supporting a null effect.* ***Abbreviations:*** *Adj., adjusted; C, cluster; CrI, credible interval; DPM, Dirichlet process mixture model; E[K], expected number of clusters; P, probability; RE, random effects; RoB, risk of bias; RR, risk ratio; TIS, Teratology Information Service; α, concentration parameter; τ, between-study heterogeneity standard deviation; ΔRR, change in risk ratio.*

**Supplementary Table 6:** Bivariate Meta-Analysis of Joint Modeling of Major Congenital Malformations and Spontaneous Abortion Using Gaussian Copula.

| **Analysis Component** | **Parameter/Study** | **Prior/Model** | **Estimate** | **Result (95% CrI)** | **Interpretation** |
| --- | --- | --- | --- | --- | --- |
| Study Data | Panchaud 2013 – MCM | — | 5/167 vs 9/341 | RR = 1.13 | Exposed vs control events/n |
|  | Panchaud 2013 – SAB | — | 30/235 vs 56/444 | RR = 1.01 | Exposed vs control events/n |
|  | Loureiro 2005 – MCM | — | 2/94 vs 9/368 | RR = 0.87 | Exposed vs control events/n |
|  | Loureiro 2005 – SAB | — | 6/106 vs 25/389 | RR = 0.88 | Exposed vs control events/n |
| Univariate Marginal | MCM pooled | μ ~ N(0, 1); τ ~ HC(0, 0.5) | RR = 1.01 | 0.35–2.60 | τ = 0.430 |
|  | SAB pooled | μ ~ N(0, 1); τ ~ HC(0, 0.5) | RR = 0.97 | 0.43–1.94 | τ = 0.428 |
| Bivariate Model Priors | Effect size (MCM) | μ_MCM ~ Normal(0, 1) | — | — | Weakly informative, centered on null |
|  | Effect size (SAB) | μ_SAB ~ Normal(0, 1) | — | — | Weakly informative, centered on null |
|  | Heterogeneity (MCM) | τ_MCM ~ Half-Cauchy(0, 0.5) | — | — | Allows small-moderate heterogeneity |
|  | Heterogeneity (SAB) | τ_SAB ~ Half-Cauchy(0, 0.5) | — | — | Allows small-moderate heterogeneity |
|  | Correlation (ρ) | ρ ~ Uniform(-0.9, 0.9) | — | — | Gaussian copula linking outcomes |
| Bivariate Model Results | MCM pooled (joint) | Gaussian copula | RR = 1.03 | 0.36–2.66 | τ = 0.445 (0.000–1.338) |
|  | SAB pooled (joint) | Gaussian copula | RR = 0.98 | 0.49–1.89 | τ = 0.342 (0.000–1.038) |
|  | Correlation (ρ) | MCM-SAB correlation | ρ = -0.005 | -0.812 to 0.898 | Near-zero correlation; outcomes independent |
| Sensitivity Analysis | Prior 1: ρ ~ Uniform(-0.9, 0.9) | Wide prior (default) | ρ = -0.005 | -0.812 to 0.898 | MCM RR = 1.03; SAB RR = 0.98 |
|  | Prior 2: ρ ~ Uniform(-0.5, 0.3) | Constrained (negative expected) | ρ = -0.101 | -0.475 to 0.283 | MCM RR = 1.02; SAB RR = 0.98 |
|  | Prior 3: ρ = 0 (fixed) | Independent outcomes | ρ = 0.000 | — | MCM RR = 1.02; SAB RR = 0.97 |
| Model Comparison | Univariate vs Bivariate (MCM) | ΔRR | Δ = 0.01 | — | Minimal difference; joint modeling stable |
|  | Univariate vs Bivariate (SAB) | ΔRR | Δ = 0.01 | — | Minimal difference; joint modeling stable |
|  | Correlation significance | ρ includes 0? | Yes | CrI spans zero | MCM and SAB statistically independent |

***Notes:*** *The bivariate copula model jointly estimated effects on MCM and SAB while accounting for potential correlation between these competing pregnancy outcomes; the estimated correlation was near zero (ρ = -0.005) with wide credible intervals spanning both negative and positive values, indicating that MCM and SAB were statistically independent in this analysis; pooled estimates were robust across all correlation prior specifications, with MCM RR ranging from 1.02 to 1.03 and SAB RR consistently at 0.97–0.98; only two studies (Panchaud 2013, Loureiro 2005) reported both outcomes and were included in the bivariate analysis.* ***Abbreviations:*** *CrI, credible interval; HC, Half-Cauchy distribution; MCM, major congenital malformations; N, normal distribution; RR, risk ratio; SAB, spontaneous abortion; τ, between-study heterogeneity standard deviation; ρ, correlation coefficient between outcomes; Δ, difference.*
